# Evaluating kidney function using a point-of-care creatinine test in Ugandan children with severe malaria: a prospective cohort study

**DOI:** 10.1186/s12882-021-02573-x

**Published:** 2021-11-06

**Authors:** Anthony Batte, Kristin J. Murphy, Ruth Namazzi, Katrina Co, Robert O. Opoka, John M. Ssenkusu, Chandy C. John, Andrea L. Conroy

**Affiliations:** 1 Child Health and Development Centre, Makerere University College of Health Sciences, Kampala, Uganda; 2 Department of Pediatrics, Ryan White Center for Pediatric Infectious Disease and Global Health, Indiana University School of Medicine, 1044 W. Walnut St., Indianapolis, IN 46202 USA; 3 Department of Paediatrics and Child Health, Makerere University College of Health Sciences, Kampala, Uganda; 4 Department of Epidemiology and Biostatistics, Makerere University School of Public Health, Kampala, Uganda

**Keywords:** Acute kidney injury, Diagnosis, Point-of-care testing, Sub-Saharan Africa, Prevalence, Mortality, Pediatric, Malaria

## Abstract

**Background:**

Acute kidney injury (AKI) disproportionately affects individuals in low-and middle-income countries (LMIC). However, LMIC—particularly countries in sub-Saharan Africa— are under-represented in global AKI research. A critical barrier in diagnosing AKI is access to reliable serum creatinine results. We evaluated the utility of a point-of-care test to measure creatinine and diagnose AKI in Ugandan children with malaria.

**Methods:**

Paired admission creatinine was assessed in 539 Ugandan children 6 months to 4 years of age hospitalized with severe malaria based on blood smear or rapid diagnostic test. Creatinine levels were measured using isotope dilution mass spectrometry (IDMS)-traceable methods. The reference creatinine was measured using the modified Jaffe method by a certified laboratory and the point-of-care testing was conducted using an i-STAT blood analyzer (i-STAT1, with and without adjustment for the partial pressure of carbon dioxide). AKI was defined and staged using the Kidney Disease: Improving Global Outcomes criteria.

**Results:**

The mean age of children was 2.1 years, and 21.6% of children were stunted. Mortality was 7.6% in-hospital. Over the entire range of measured creatinine values (<0.20mg/dL-8.4mg/dL), the correlation between the reference creatinine and adjusted and unadjusted point-of-care creatinine was high with R^2^ values of 0.95 and 0.93 respectively; however, the correlation was significantly lower in children with creatinine values <1mg/dL (R^2^ of 0.44 between the reference and adjusted and unadjusted i-STAT creatinine). The prevalence of AKI was 45.5% using the reference creatinine, and 27.1 and 32.3% using the unadjusted and adjusted point-of-care creatinine values, respectively. There was a step-wise increase in mortality across AKI stages, and all methods were strongly associated with mortality (*p*<0.0001 for all). AKI defined using the reference creatinine measure was the most sensitive to predict mortality with a sensitivity of 85.4% compared to 70.7 and 63.4% with the adjusted and unadjusted point-of-care creatinine values, respectively.

**Conclusions:**

Point-of-care assessment of creatinine in lean Ugandan children <4 years of age underestimated creatinine and AKI compared to the clinical reference. Additional studies are needed to evaluate other biomarkers of AKI in LMIC to ensure equitable access to AKI diagnostics globally.

## Background

Acute kidney injury (AKI) is an important public health challenge, particularly in low-and middle-income countries (LMIC) where 85% of the estimated 13.3 million cases per year occur [1, 2]. AKI is widely recognized as a risk factor for mortality with large multi-site international studies in neonates [3], children [4] and adults [5] all identifying AKI as an independent risk factor for mortality. LMIC are critically under-represented in global AKI research—particularly sub-Saharan Africa—in part due to limitations in the availability, accessibility and affordability of diagnostic tools [6].

Mortality in AKI in children in sub-Saharan Africa remains unacceptably high at 34% compared to a global average of 14% [7]. Failure to recognize AKI can lead to disease progression requiring more aggressive therapies like kidney replacement therapy that are costly and often unavailable in LMIC. Early recognition of children at risk of AKI and the initiation of appropriate supportive care to correct hypovolemia and discontinue the use of non-essential nephrotoxic medications can reduce progression and improve outcomes [8]. Malaria is a significant cause of AKI in sub-Saharan Africa [9–14], and malaria-associated AKI is associated with substantial morbidity and mortality [15–19], increasing the risk of mortality in-hospital as well as an increased long-term risk of neurocognitive and behavioral problems in survivors [18, 20].

AKI is diagnosed based on increases in serum creatinine (SCr) or decreases in urine output [21]. In practice, AKI diagnosis is often based on changes in SCr as urine output is challenging to accurately quantify in critically ill populations without catheterization and is further complicated by insufficient nursing care in many LMIC. In LMIC, AKI is primarily a community-acquired condition affecting previously healthy children and young adults on admission to the hospital [22]. Therefore, SCr is the most useful measure in the initial assessment of AKI in a febrile child presenting with dehydration or volume depletion. To improve awareness and recognition of AKI as an important complication in children in LMIC, clinicians need access to tools to accurately measure kidney function [6, 22].

Although children with severe malaria frequently present with multiple severe malaria complications and multi-organ dysfunction [18, 23], AKI is a common complication in children with severe malaria [17, 18] and independently predicts mortality [17–19, 23]. Diagnosis of AKI is dependent on the use of an appropriate estimate of baseline SCr [24] and an accurate measure of SCr. Access to SCr testing remains a critical barrier in many African health care settings [25], and increased availability of point-of-care devices to measure SCr across all levels of health care could substantially improve the recognition and management of AKI in resource-limited settings. The majority of studies assessing performance of point-of-care devices to measure SCr have been conducted in high-income settings in adult populations [26–28] where SCr levels are substantially higher than pediatric populations. Therefore, there is a need to evaluate point-of-care tests in pediatric populations where small changes in SCr may constitute AKI.

We evaluated the performance of the i-STAT1 handheld blood analyzer as a point-of-care tool to measure SCr and define AKI compared to a reference value obtained by a certified clinical laboratory. We present analyses comparing the correlation, bias, and precision of measures over the entire range of SCr values obtained. Further, as our population of interest is children <5 years of age with low estimated baseline SCr values, we conducted additional analyses focusing on the lower range of SCr values characteristic of lean pediatric populations. Using the SCr values from the different test methods, we assessed the prevalence and severity of AKI on admission and evaluated the ability of the different AKI definitions to predict mortality.

## Methods

### Study population

Between 2014 and 2017, 600 children with a clinical definition of severe malaria were enrolled in the study. Children were eligible if they were between 6 months to 4 years of age, had diagnostic evidence of malaria with either a positive rapid diagnostic test for *Plasmodium falciparum* histidine-rich protein-2 (HRP-2) or direct visualization of parasites by Giemsa microscopy, required hospitalization and met selected criteria for severe malaria (coma, respiratory distress, multiple seizures, severe anemia, or prostration). Exclusion criteria included known chronic illness requiring medical care, history of coma, head trauma, known developmental delay, cerebral palsy or prior hospitalization for malnutrition.

### Creatinine testing

At enrollment, all children had a venous blood draw. The i-STAT System was used to assess a basic metabolic panel (CHEM8+ cartridge: SCr, blood urea nitrogen (BUN), glucose, ionized calcium, sodium, potassium, chloride, bicarbonate) and blood gases (CG4 cartridge: pH, PCO_2_, PO_2_, TCO_2_, HCO_3_, base excess, sO_2_) on lithium heparin whole blood (Abbott Point of Care Inc., Princeton, NJ). Blood was tested immediately upon collection by trained nurses and medical officers following the manufacturer's recommendations. The i-STAT system is designed for *in vitro* quantification of SCr in arterial, venous, or capillary whole blood using 95uL of sample and provides results within two minutes with a reportable range for SCr of 0.20-20.0 mg/dL. SCr was measured using an enzymatic assay with amperometric detection on a platinum electrode. As the partial pressure of carbon dioxide (*P*CO_2_) can affect measurement of SCr by i-STAT, values were adjusted based on the *P*CO_2_ values as follows: for SCr values below 2 mg/dL and *P*CO2 values above 40 mmHg, [Cr]corrected = [Cr]i-STAT X {1 - (0.069 X [(*P*CO_2_ -40)/10])}; and for SCr values above 2 mg/dL, [Cr]corrected = [Cr]i-STAT X {1 - (0.037 X [(40 - *P*CO_2_)/10])} [29].

Serum samples were shipped to the United States of America on dry ice and SCr was measured using a Beckman Coulter AU5822 chemistry analyzer using the modified Jaffe colorimetric method (Beckman Coulter, Brea, California) at the Indiana University Pathology Laboratory. The limit of detection of the assay is 0.20 mg/dL, and the coefficient of variation of the assay is 4.1% at 0.80 mg/dL and 2.1% at 5.5 mg/dL.

Both methods of SCr measurement were traceable to an isotope dilution mass spectrometry (IDMS) reference method using the U.S. National Institute of Standards and Technology (NIST) Standard Reference material SRM967. Samples below the limit of detection were assigned a value of 0.19 mg/dL.

### Defining acute kidney injury

Acute kidney injury was defined using the Kidney Disease: Improving Global Outcomes (KDIGO) criteria [21] based on a 1.5-fold increase in SCr over the estimated baseline. Staging was as follows: stage 1, 1.5-1.9 fold increase in SCr over baseline; stage 2, 2.0-2.9 fold increase over baseline; stage 3, ≥3.0 fold increase over baseline. Baseline SCr was estimated using a height-independent approach to back-calculate SCr using the Pottel age-based equation, where eGFR=107.3/(SCr/Q), assuming a normal GFR of 120mL/min per 1.73m^2^ (height-independent), and Q=0.0270*age + 0.2329 [30]. This method was previously validated in Ugandan children and was the most accurate method to estimate baseline SCr with minimal bias [24]. A single SCr measure was available on admission, and data on urine output were not collected. Dialysis was not available on-site at the time the study was conducted.

### Statistical analysis

Data were analyzed using STATA v14.0 (StataCorp) and GraphPad Prism v7.03. Differences in SCr measures by test methodology were evaluated using a number of statistical tests. Concentrations of SCr were compared using Pearson’s correlation. The Bland-Altman method [31] was used to measure agreement between the SCr values obtained by each test approach based on the variance of differences in concentration across the mean of concentration values. Bias was represented by the mean difference between methods of estimating SCr, and precision represents one standard deviation of the bias. Proportional bias is represented by the slope of the regression line of the differences between reference and i-STAT SCr against the average of reference and i-STAT SCr, where a slope of 0 means no proportional bias. To evaluate the relationship between AKI and mortality, we used logistic regression to estimate the odds ratio (OR) adjusting for age, sex, and height-for-age z score. A *p* value <0.05 was considered significant. The ability of AKI to discriminate between children who survived and children who died was assessed using non-parametric receiver operating characteristic (ROC) curve analysis using the method of Delong et al. [32], and presenting the area under the curve.

### Role of the funding source

The funders had no role in the study design, analysis, or decision to publish.

## Results

**Fig. 1:**
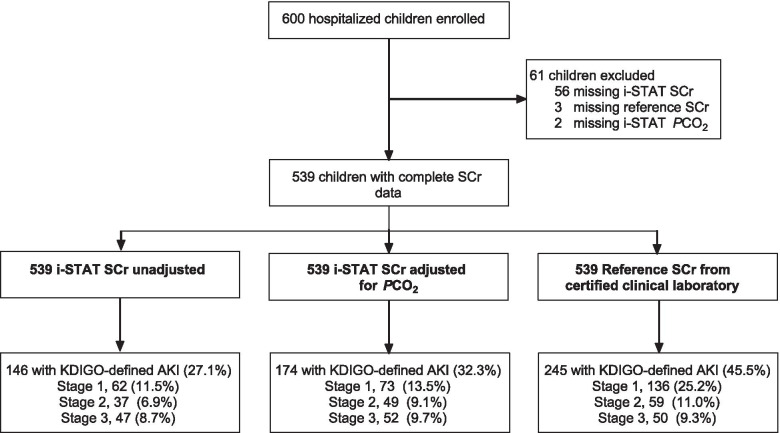
Flow chart of the study population. Between 2014 and 2017, 600 children hospitalized with evidence of malaria were enrolled in a prospective cohort study that enrolled children from Mulago National Referral Hospital in Kampala or Jinja Regional Referral Hospital in Jinja, Uganda. Acute kidney injury was defined and staged using the Kidney Disease: Improving Global Outcomes (KDIGO) criteria based on a fold change in SCr from estimated baseline. AKI was defined using the values obtained from the i-STAT handheld analyzer with or without adjustment for the partial pressure of carbon dioxide (*P*CO_2_) as well as the reference SCr from a certified clinical laboratory. The prevalence reported represents the number of children with KDIGO-defined AKI for each measure out of the 539 children with paired SCr values

Of the 600 enrolled in the study, 539 children had paired SCr measures using the point-of-care i-STAT test and by the reference laboratory and are included in this analysis. The mean (SD) age of the 539 children enrolled in this study was 2.1 (0.9) years, with 42.9% of children female. The mean anthropometric z scores were -1.1 (1.1), -1.1 (1.3), and -0.7 (1.1) for weight-for-age, height-for-age and weight-for-height respectively. Undernutrition was common, with 116 (21.6%) of children stunted on enrollment. Overall, 11 children were living with HIV-1 (2.1%), and 21 (4.0%) children had a positive blood culture for bacteremia. Although all children enrolled were diagnosed with severe malaria based on a positive blood smear or a positive rapid diagnostic test for HRP-2, only 368 (68.3%) of children included in this analysis were smear-positive for malaria. A total of 41 children died during hospitalization (7.6%). The study flow is shown in Fig. 1.

### Correlation of i-STAT vs. biochemistry SCr measures

**Fig. 2:**
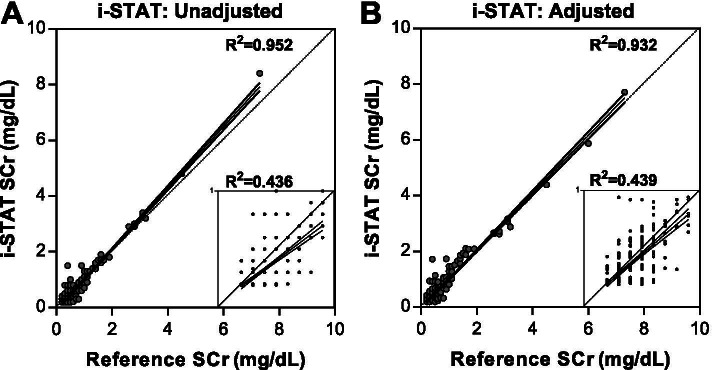
Correlation between SCr measured by the point-of-care test and the clinical reference. Scatter plot showing the SCr measures by the point-of-care and the reference SCr where (**A**) is unadjusted i-STAT value and (**B**) adjusted SCr. The R^2^ for the correlation is presented on the graph with the regression line and 95% confidence interval shaded in grey. A separate correlation was performed in children with a reference SCr value less than 1mg/dL (*n*=509) and presented as an inset in each graph

We compared the paired SCr measures from i-STAT (adjusted and unadjusted for *P*CO_2_) to the reference SCr from a clinical laboratory. The mean (SD) of the reference SCr value was 0.49 (0.56), and the unadjusted and adjusted i-STAT SCr values were 0.44 (0.63) and 0.47 (0.60), respectively. The values ranged from 0.19 mg/dL as the lowest value across all assays to the highest value of 7.3 mg/dL for the reference SCr, 8.4 mg/dL for the unadjusted i-STAT SCr, and 7.7 mg/dL for the adjusted i-STAT SCr. Overall, the correlation between the reference and point-of-care methods was high over the entire range of the assay with R^2^ values of 0.95 and 0.93 for the unadjusted and adjusted SCr values, respectively (Fig. 2). When the analysis was restricted to children with a SCr measure <1mg/dL by the reference test (*n*=504, 94% of all results), the correlation was significantly lower with an R^2^ value of 0.44 between the reference SCr and both the unadjusted and adjusted point-of-care SCr measures (Fig. 2).

### Agreement between SCr from the clinical reference laboratory and point-of-care i-STAT

**Fig. 3:**
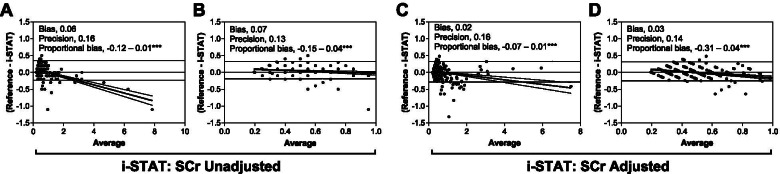
Bland-Altman analysis comparing the agreement between point-of-care SCr and the clinical reference. A Bland-Altman graph plotting the difference between values on the y-axis (Reference- i-STAT) compared to the average value [(Reference + i-STAT)/2] over the range of the assay (**A, C**) and when the reference SCr measure was <1 mg/dL (**B, D**). The bias represents the absolute mean difference between the reference and i-STAT SCr measure, and the precision is one standard deviation of the bias. The horizontal dashed line depicts the 95% limits of agreement. Proportional bias was evaluated by testing if the slope of the linear regression model of the difference between reference and i-STAT SCr against the average of reference and i-STAT SCr differed from zero. Proportional bias represents the slope (B1) + SE (standard error), and the asterisks indicate whether the slope is statistically different from zero. ***p<0.0001

To assess agreement between the point-of-care and clinical reference concentrations of SCr, we used the Bland-Altman method. This graphical tool can present systematic bias between two methods of measurement and identify outliers across the range of the assay (Fig. 3). We conducted the Bland-Altman analysis over the entire range of SCr measures obtained and also focused on evaluating the bias and precision of the assay at the lower range of the assay (SCr <1 mg/dL) where the majority of SCr measures fell. Compared to the reference value, there was more bias with lower SCr measures by the point-of-care test with the largest difference observed when the values were not corrected for the *P*CO_2_. Proportional bias was assessed by evaluating whether the slope of the regression line presented on the graphs was different from zero. Irrespective of whether i-STAT measures were adjusted or unadjusted, there was significant proportional bias (Fig. 3). Although the estimated baseline SCr value varied by age, the mean estimated baseline for the study population was 0.26mg/dL (range, 0.22- 0.30). The mean and range of SCr across AKI stages using the reference SCr measure was as follows: stage 1 AKI, mean 0.44 mg/dL (range: 0.34-0.57 mg/dL); stage 2 AKI, mean 0.60 mg/dL (range: 0.48-0.82 mg/dL); stage 3 AKI, mean 1.7 mg/dL (range: 0.72-7.3 mg/dL).

### The relationship between AKI and mortality by SCr measure

**Fig. 4:**
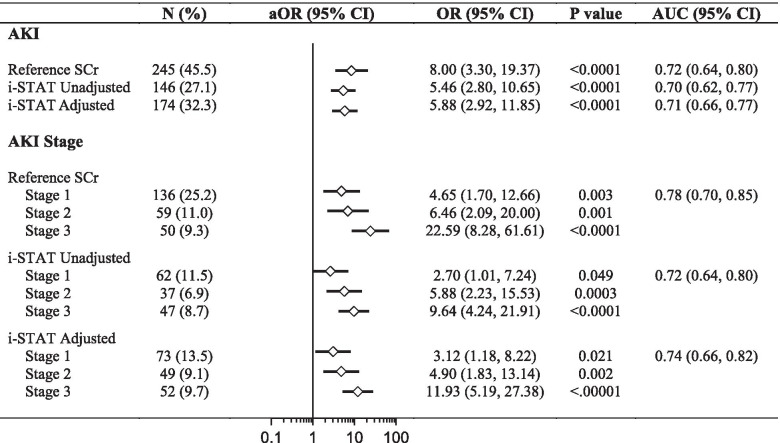
Forest plot showing the relationship between AKI and mortality. Table showing the frequency of KDIGO-defined AKI and AKI stage using the different methods to measure serum SCr (SCr) with the corresponding odds ratio (OR) and 95% confidence interval (CI) from logistic regression models. The forest plot depicts the adjusted OR (aOR) from a logistic regression model adjusting for child age, sex, and height-for-age z score. The ability of the different AKI definitions to discriminate between children who died or survived was assessed using receiver operating characteristic curve analysis, and the area under the curve (AUC) and 95% CI are presented

As AKI is well-recognized as a predictor of mortality, we evaluated the ability of AKI to discriminate between children who survived and children who died as an objective and clinically meaningful outcome (Fig. 4). Using ROC curve analysis to evaluate the ability of the different measures to discriminate between the hospital outcome, all AKI definitions had moderate, and comparable, discriminatory ability with the areas under the curve (AUC) ranging from 0.70-0.72 (Fig. 4). AKI defined using the reference SCr measure was the most sensitive to predict mortality with a sensitivity of 85.4% compared to 70.7% with the adjusted point-of-care SCr and 63.4% with the unadjusted point-of-care SCr. When AKI was staged, the AUC for the reference laboratory (0.78) was higher than the AUC from the point-of-care SCr (0.72-0.74). The unadjusted point-of-care SCr underestimated Stage 1 and Stage 2 AKI compared to the reference method (Stage 1, 25.2% (reference) vs. 11.5% (i-STAT unadjusted); Stage 2, 11.0% (reference) vs. 6.9% (adjusted point-of-care SCr), which resulted in lower sensitivity to predict mortality (Fig. 4).

By multivariate analysis, AKI remained an independent predictor of mortality following adjustment for child age, sex, and nutritional status (height-for-age z score). These findings were consistent across AKI definitions and AKI stages irrespective of the method to measure SCr (Fig. 4), and there was a step-wise increase in mortality across AKI stages.

## Discussion

AKI is an under-recognized clinical complication associated with significant pediatric mortality globally. In the present study, we evaluated the ability of the i-STAT handheld point-of-care blood analyzer to measure SCr compared to a clinical reference using the modified Jaffe reaction. Compared to the certified clinical laboratory, the point-of-care test measured lower SCr values even after adjustment for the partial pressure of CO_2_ that is recognized to affect test performance. While the correlation between the two assays was high over the whole range of reported values (0.19-8.4 mg/dL), there was only moderate correlation across the range of lower SCr values typically measured in young children. When AKI was defined based on the unadjusted point-of-care SCr compared to the clinical reference, 99 (40%) of AKI episodes were missed (99/245). Although AKI was strongly associated with mortality across all AKI definitions with a step-wise increase in mortality across AKI stages, the relationship between AKI and mortality was strongest in children with SCr measured by the certified clinical laboratory.

The reference method employed in this study was measuring SCr using the modified Jaffe reaction. Although the modified Jaffe reaction is recognized as traceable to the international standard, it is susceptible to interference through pseudo-chromogens resulting in SCr overestimation. Enzymatic reactions, like the ones used by the handheld i-STAT device, are generally considered more sensitive and specific than the Jaffe reaction. However, enzymatic SCr tests are considerably more expensive (estimated at $2.00 per test rather than $0.30) than the modified Jaffe reaction [33]. Thus, the enzymatic test is not offered by all laboratories, was not available through the clinical laboratory that performed the testing, and is not currently available in Uganda. Therefore, while the reference standard remains an imperfect standard, the modified Jaffe reaction remains the only laboratory method accessible in Uganda.

Although i-STAT SCr correlated strongly with the reference measure across the entire range of the assay, the correlation was only moderate in children with a SCr value <1 mg/dL. As this was a relatively young pediatric population and 21.5% of children were stunted according to WHO standards, the estimated baseline SCr was low across the population, so slight fluctuations in SCr had a relatively large impact on the estimated prevalence of AKI, reflecting limitations in SCr-based AKI definitions. Correspondingly, the AKI definitions differed in their ability to predict mortality. The unadjusted i-STAT SCr measure had a sensitivity of 63.4% to predict mortality compared to 85.4% using the reference method to define AKI. Although the sensitivity of the assay was better when adjusting for the *P*CO_2_, the requirement for an additional cartridge to obtain the *P*CO_2_ measure makes it considerably more expensive to obtain adjusted values and impractical in a clinical setting.

Although the i-STAT had lower sensitivity to define AKI in this pediatric population, it remained strongly associated with mortality (mortality, 3.8% in children without i-STAT defined AKI compared to 17.8% in children with i-STAT defined AKI), and was able to correctly identify 41/50 (82%) of children with Stage 3 AKI where mortality was 32.0%. Thus, i-STAT remains a valuable point-of-care tool to identify children with AKI at increased risk of death in settings where laboratory assessment of SCr is unavailable. The i-STAT system would likely have better performance in adult populations where baseline SCr values are considerably higher, and identifying a 50% change in SCr from baseline would be less affected by assay imprecision. Most of the discrepancies in AKI in the present study were in defining Stage 1 AKI, which represented an average increase in SCr from an estimated baseline from 0.26mg/dL to 0.44 mg/dL.

In a global survey published in 2017 to assess the current state of kidney care, only 12% of countries in Africa reported that they usually have the services to measure SCr with eGFR reporting at the primary care level, while 39% reported they never have the services available [25]. Further, when assessing reported methods of SCr assessment in sub-Saharan Africa, most papers (82%) do not report whether SCr was measured using IDMS traceable assays, and only 6/80 studies used the more accurate enzymatic method [34]. This highlights the inherent challenges in measuring SCr across sub-Saharan and the need for substantial development of laboratory capacity in order to expand access to SCr testing. As SCr is a relatively late marker of AKI, is affected by nutritional status and age, and is logistically and technically challenging to measure, efforts to identify and validate alternative biomarkers of AKI are needed.

Although it is not specific to AKI, blood urea nitrogen (BUN) accumulates when kidney function decreases. In Ugandan children with severe malaria, BUN is strongly correlated with AKI and outperforms serum SCr in predicting mortality [17]. The prognostic utility of BUN has been demonstrated in over 5000 African children with severe malaria across 11 sites in 9 African countries [35]. BUN is water-soluble and can be monitored non-invasively in saliva (saliva urea nitrogen, SUN) using a semi-quantitative lateral flow dipstick. Studies assessing the discriminative ability of dipstick SUN to detect elevated BUN have reported sensitivities ranging from 77-85% and specificities of 85-88% [36]. SUN has been tested in several adult populations, including populations in low-resource settings, where SUN had good performance of identifying AKI with areas under the ROC curve >0.80 [37–40]. Recent validation in a pediatric population from Sudan found that SUN could be used to identify, screen, and monitor pediatric patients with severe AKI [41].

A handheld point-of-care device that could be employed across all levels of health care would present an opportunity to substantially improve the recognition and management of AKI in resource-limited settings. In order for devices to be useful, they must be able to measure kidney function accurately, affordably, and should be amenable for use in remote tropical settings where heat and humidity can affect test performance [36]. For example, the upper limit of the temperature range for the i-STAT is 30°C; however, many regions in sub-Saharan Africa have temperatures that exceed 30^o^C during hot seasons. Further, as i-STAT machines are not widely available in sub-Saharan Africa, it can be expensive and logistically challenging to maintain a supply of cartridges and controls that are not expired. Shelf-life is an important consideration when assessing the cost and performance of different platforms, and the ability to operate without a need for electricity in sites where there are frequent interruptions to grid electricity. A recent review assessed the strengths and limitations of existing point-of-care platforms for use in LMIC [36]. All test platforms cost thousands of dollars to purchase the device (range:~ $3995-20,000 USD), and test cartridges or strips cost in the range of $4-20 USD per test [36]. Without significant reductions in price to increase accessibility, existing point-of-care SCr tests are not appropriate for routine clinical use in LMIC settings.

While a diagnosis of AKI currently requires a measure of SCr or assessment of urine output, other measures of impaired kidney function may have utility in identifying children with kidney dysfunction in LMIC. Additional studies are needed to evaluate the performance of existing AKI biomarkers (e.g., urine neutrophil gelatinase-associated lipocalin [42–44], Cystatin C [44–47], SUN [37–40]), as well as next-generation AKI biomarkers [44, 45, 48] in LMIC settings where the etiology and pathophysiology of AKI may differ from high-income settings. As AKI biomarkers are discovered and validated, it is crucial to consider their utility across all resource settings.

## Conclusion

In this study, we found that the i-STAT measurement of SCr in a population of lean Ugandan children underestimated SCr compared to a clinical reference measure and led to the under-diagnosis of AKI. AKI was strongly associated with mortality irrespective of test methodology. The development of point-of-care approaches to measure SCr with good precision at lower SCr concentrations would be beneficial for pediatric populations. More importantly, there is an urgent need for AKI diagnostics that are temperature stable, affordable, and can be equitably accessed by patients in LMIC.

## Data Availability

The datasets used and/or analyzed during the current study are available from the corresponding author on reasonable request.

## Abbreviations

AKI: Acute Kidney Injury
AUC: Area under the curve
BUN: Blood urea nitrogen
CI: Confidence Interval
GFR: Glomerular filtration rate
HIV: Human immunodeficiency virus
KDIGO: Kidney Disease: Improving Global Outcomes
LMIC: Low- and middle-income country
NGAL: Neutrophil gelatinase-associated lipocalin
OR: Odds ratio
*P*CO_2_: Partial pressure of carbon dioxide
ROC: Receiver operator characteristic
SCr: Serum creatinine
SUN: Saliva urea nitrogen
WHO: World Health Organization
